# The zebrafish genome encodes the largest vertebrate repertoire of functional aquaporins with dual paralogy and substrate specificities similar to mammals

**DOI:** 10.1186/1471-2148-10-38

**Published:** 2010-02-11

**Authors:** Angèle Tingaud-Sequeira, Magdalena Calusinska, Roderick N Finn, François Chauvigné, Juanjo Lozano, Joan Cerdà

**Affiliations:** 1 Laboratory of Institut de Recerca i Tecnologia Agroalimentàries (IRTA)-Institut de Ciències del Mar, Consejo Superior de Investigaciones Científicas (CSIC), 08003 Barcelona, Spain; 2 Génomique et Physiologie des Poissons, Université Bordeaux 1, UMR NuAGe, 33405 Talence, France; 3 Center for Protein Engineering, University of Liège, B-4000 Liège, Belgium; 4 Department of Biology, University of Bergen, Bergen High Technology Center, N-5020 Bergen, Norway; 5 Centro de Investigación Biomédica en Red de Enfermedades Hepáticas y Digestivas (CIBERehd), 08036 Barcelona, Spain

## Abstract

**Background:**

Aquaporins are integral membrane proteins that facilitate the transport of water and small solutes across cell membranes. These proteins are vital for maintaining water homeostasis in living organisms. In mammals, thirteen aquaporins (AQP0-12) have been characterized, but in lower vertebrates, such as fish, the diversity, structure and substrate specificity of these membrane channel proteins are largely unknown.

**Results:**

The screening and isolation of transcripts from the zebrafish (*Danio rerio*) genome revealed eighteen sequences structurally related to the four subfamilies of tetrapod aquaporins, i.e., aquaporins (AQP0, -1 and -4), water and glycerol transporters or aquaglyceroporins (Glps; AQP3 and AQP7-10), a water and urea transporter (AQP8), and two unorthodox aquaporins (AQP11 and -12). Phylogenetic analyses of nucleotide and deduced amino acid sequences demonstrated dual paralogy between teleost and human aquaporins. Three of the duplicated zebrafish isoforms have unlinked loci, two have linked loci, while DrAqp8 was found in triplicate across two chromosomes. Genomic sequencing, structural analysis, and maximum likelihood reconstruction, further revealed the presence of a putative pseudogene that displays hybrid exons similar to tetrapod AQP5 and -1. Ectopic expression of the cloned transcripts in *Xenopus laevis *oocytes demonstrated that zebrafish aquaporins and Glps transport water or water, glycerol and urea, respectively, whereas DrAqp11b and -12 were not functional in oocytes. Contrary to humans and some rodents, intrachromosomal duplicates of zebrafish AQP8 were water and urea permeable, while the genomic duplicate only transported water. All aquaporin transcripts were expressed in adult tissues and found to have divergent expression patterns. In some tissues, however, redundant expression of transcripts encoding two duplicated paralogs seems to occur.

**Conclusion:**

The zebrafish genome encodes the largest repertoire of functional vertebrate aquaporins with dual paralogy to human isoforms. Our data reveal an early and specific diversification of these integral membrane proteins at the root of the crown-clade of Teleostei. Despite the increase in gene copy number, zebrafish aquaporins mostly retain the substrate specificity characteristic of the tetrapod counterparts. Based upon the integration of phylogenetic, genomic and functional data we propose a new classification for the piscine aquaporin superfamily.

## Background

Aquaporins constitute a superfamily of major intrinsic proteins (MIPs) that facilitate passive, yet remarkably efficient permeation of water molecules across cellular membranes [1,2]. Some aquaporins can also permeate non-ionic compounds, such as glycerol and urea, and are termed glycerol facilitators (Glps) or aquaglyceroporins. The first water channel was isolated from human red blood cell membranes as a novel integral membrane protein of 28 kDa (CHIP28). This channel is now termed aquaporin-1 (AQP1) [3]. Subsequently, thirteen aquaporin paralogs (AQP0-12) have been identified in mammals [1], and up to 38 MIP-related sequences, divided into four types, plasma membrane intrinsic proteins (PIPs), tonoplast intrinsic proteins (TIPs), small and basic intrinsic proteins (SIPs) and nodulin 26-like intrinsic proteins (NIPs), have been documented in plants [4-6]. Each form is composed of a single polypeptide chain varying in length from ~270-350 amino acids that spans the lipid bilayer six times with three extracellular loops (loop A, C and E) and two intracellular loops (loop B and D), which have their N- and C-termini located intracellularly. The loops B and E fold and extend intramembranous hemi-helices that bear the highly conserved amino acid motifs Asn-Pro-Ala (NPA), which are involved in the formation of the water pore. When translocated to the cell membrane, most aquaporins form homotetramers [7-9], in which one or two monomers may be glycosylated, but each monomer functions as an independent water channel [10,11].

The maintenance of body fluid homeostasis is essential for the survival of any living organism. Aquatic animals also face the problem of direct interaction with their aqueous environment. Depending upon the life history of the species, the osmolarity of the saline or freshwater medium differs manifold from their internal body fluids, and therefore an important physiological role of aquaporins would be expected. Accordingly, some studies in teleosts have reported differential mRNA and protein expression of specific aquaporin isoforms in osmoregulatory organs in response to changes in environmental salinity [12-16]. Moreover, recent studies of neofunctionalized vitellogenins and the essential role of an AQP1-related channel have revealed the evolutionary importance of oocyte hydration in marine teleosts as a pre-adaptation to spawning in the hyper-osmotic oceanic environment [17-24]. To date, however, the genomic repertoire of aquaporin isoforms present in teleosts, as well as the permeability properties of the encoded proteins, remains largely unknown.

During the last decades, the zebrafish (*Danio rerio*) has become a powerful model organism in comparative genomics and developmental biology. This species is amenable for genetic analysis in which large-scale mutagenesis screens have been successfully performed, and a large amount of genomic and expressed sequence tags (ESTs) data, BAC libraries and fine genetic linkage maps have been accumulated [25]. Work with zebrafish has also extended its application to a wide variety of experimental studies relevant to human disease, such as cardiovascular disorders, angiogenesis and neurological and renal diseases [26,27]. Aquaporins have been shown to play important roles in some of these alterations [28], and therefore the diversity and functional properties of zebrafish aquaporins need to be determined prior to using this species as a suitable experimental model in biomedical research and comparative physiology.

With the sequence of the zebrafish genome completed, the complexity of the teleost aquaporin gene family can be assessed. In the present study, we screened the zebrafish genome for aquaporin-related sequences and determined their phylogenetic relationships, permeability properties, and the pattern of mRNA expression in adult tissues. We found a high number of functional aquaporins in this species, and based upon the data obtained we propose a new classification of the piscine aquaporin superfamily.

## Results and Discussion

### The zebrafish aquaporin gene family

**Figure 1:**
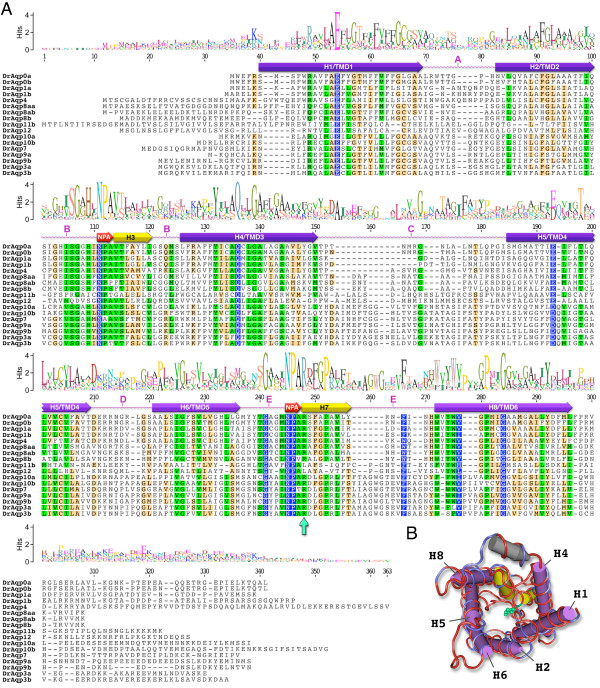
**Amino acid sequence alignment of zebrafish aquaporins**. (A) The consensus sequence logo is scaled according to amino acid conservation. Highest residue similarity (blue: 100%, green: 80-100% or sand; 60-80%) is found within the α-helical regions (H1-8). The transmembrane domains (TMD1-6) are annotated for DrAqp0a based upon a molecular sequence wrap to the crystallographically resolved structure of *Bos taurus *AQP0 (B). The structure wrap consists of the complete peptides (263 amino acids) with a gapless identity/similarity of 70.3/85.9%. The render shows identical residues in red, non-identical in blue. The hemi-helices H3 and H7 (yellow) on loops B and E, respectively, fold such that the opposing NPA motifs (pink in the alignment) interact to present the arginine constriction (DrAqp0a R^187 ^green ball and stick, and arrow in alignment). The C-terminal domain is shown with a grey α-helix.

The screening of the zebrafish genome revealed the presence of 18 putative members of the aquaporin superfamily, most of them existing as duplicate or triplicate genes, ranging in size from 2.2 to 18 kb that encode proteins between 255-320 amino acids long (Figure 1). We successfully cloned and characterized 17 of these transcripts. The nomenclature of these genes was chosen based on their phylogenetic position and chromosomal locus, their identity to human orthologs, as well as on their structural and functional features (see below), in accordance with the recommended guidelines for Human Genome Nomenclature [29] and the Zebrafish Information Network [30]. Thus, zebrafish aquaporin-4, -7, -11b and -12 (*draqp4*, *-7*, *-11b *and *-12*, respectively) are present as single copy genes, whereas aquaporin-0, -1, -3, -9 and -10 genes (*draqp0a*, *-0b*, *-1a*, *-1b*, *-3a*, *-3b*, *-9a*, *-9b*, *-10a *and *-10b*, respectively) are duplicates. For the *draqp8 *gene, three copies were found (*draqp8aa*, *-8ab *and *-8b*). Unlike the other aquaporin isoforms, we found that Aqp8 has both tandem and genomic duplicates encoded within the genomes of zebrafish and stickleback (*Gasterosteus aculeatus*) (see below for an explanation of the nomenclature).

**Figure 2:**
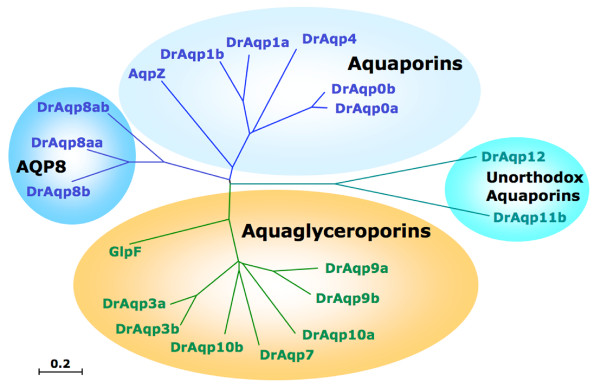
**Phylogenetic relationships among zebrafish aquaporins**. The unrooted phylogenetic tree was constructed using the NJ method. The *Escherichia coli *homologs (EcAqpZ and EcGlpF) cluster as aquaporins and aquaglyceroporins, respectively. The bar indicates the mean distance of 0.2 changes per amino acid residue.

The deduced amino acid sequences of zebrafish aquaporins contain six predicted transmembrane helices and two NPA boxes that are the hallmark of the MIP superfamily [31]. The only exception was DrAqp8b in which only five transmembrane domains could be determined *in silico*, despite the equality in sequence length. Analysis of the primary structures of the zebrafish aquaporins indicated that they could be classified into two paralogous subgroups and two more divergent subgroups (Figure 2). The first subgroup includes DrAqp0a, -0b, -1a, -1b and -4, which share 35-85% amino acid identity (Additional file 1) and clustered with the *Escherichia coli *aquaporin-Z (EcAqpZ), thus suggesting that these aquaporins belong to the water-specific aquaporin subfamily [31]. The identity of the duplicated isoforms present in this group varied, since DrAqp0a and -0b were 85% identical, whereas DrAqp1a and -1b share only 61% identity (Additional file 1). The second group includes DrAqp3a, -3b, -7, -9a, -9b, -10a and -10b, which were 40-73% identical and clustered together with the *E. coli *glycerol facilitator (EcGlpF), suggesting that they belong to the aquaglyceroporin (Glp) subfamily. In this group, the identity between DrAqp3a and -3b, DrAqp9a and -9b, and DrAqp10a and -10b, was 73, 61 and 45%, respectively. The percent identity between the aquaporin and Glp groups was as low as 19-28%. The most divergent paralogs were those including the AQP8-like (DrAqp8aa, -8ab and -8b), and DrAqp11b and -12 sequences, which share 23-28% and 13-25% identity, and 20-14% and 4-22% identity with aquaporins and Glps, respectively. DrAqp8aa, -8ab and -8b share 43-60% identity between them, and DrAqp11b and -12 were 27% identical. These observations indicate that zebrafish harbours the largest complement of aquaporin genes of any vertebrate studied to date, which can be classified into four tetrapod-like subfamilies [2]: classical water-selective aquaporins (*AQP0*, *AQP1 *and *AQP4*), a water and urea transporter (*AQP8*), classical Glps (*AQP3 *and *AQP7-10*), and two unorthodox aquaporins (*AQP11b *and *-12*).

### Genomic organization

**Figure 3:**
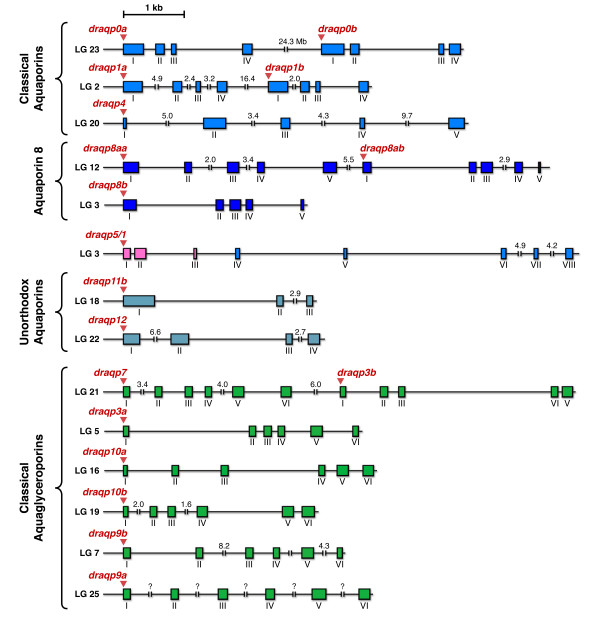
**Genomic organization of zebrafish aquaporins**. Schematic representation of zebrafish aquaporin gene structures and chromosomal loci. The boxes indicate exons with coding regions only. Distances are in kb or in Mb when indicated. In the case of *draqp9a*, the quality of the genomic sequence available was insufficient to establish the size of the introns.

The division of the zebrafish aquaporin superfamily into four subfamilies inferred from comparison of the deduced protein sequences is mirrored in the intron-exon structures (Figure 3). Most classical aquaporins include four exons, *draqp4 *being an exception with five exons, whereas the Glp genes are characterized by six exons, with the exception of *draqp3b*, which is encoded by five exons. The *draqp8aa*, *draqp8ab *and *draqp8b *transcripts are invariably coded by five exons, whereas the unorthodox *draqp12 *showed four exons as the aquaporin subfamily. In *draqp11b*, three exons were observed although in this case the intron-exon boundaries were not well defined and therefore the number of exons could not be determined with certainty. In the majority of zebrafish aquaporin genes the intron lengths were <5 kb, although the last intron of *draqp4 *was of 9.7 kb, whereas the second intron of *draqp9b *and *draqp8aa*, and the first intron of *draqp12*, were of 8.2, 6.3 and 6.6 kb in length. In the case of *draqp9a*, the quality of the genomic sequences available did not allow assessment of intron length.

Examination of the zebrafish aquaporin gene structures in relation to orthologs in other metazoan organisms revealed a generally well-conserved exon-intron organization (Additional file 2). Classical aquaporins (*aqp0*, *-1*, *-2*, *-5 *and *-6*) are encoded by 4 exons, with *Xenopus tropicalis aqp1 *as an apparent exception with 2 exons. Metazoan *aqp4 *is encoded by 5 exons, although *Drosophila melanogaster aqp4 *shows some variability. *aqp8 *is a polymorphic gene encoded by 6 exons in tetrapods, 5 exons in teleosts, 2-3 exons in Diptera and 6-7 exons in Nematoda. Classical Glps are highly conserved with 5-6 exons in metazoa, while the vertebrate-specific unorthodox *aqp11 *has 3 exons, but the more ancestral unorthodox *aqp12 *retains 3-4 exons in vertebrates, but 4-7 exons in invertebrates.

*In silico *genomic screening using ensembl v56 [32] also revealed the presence of an 18th gene in zebrafish (ENSDARG00000038202) with an anomalous structure showing 8 exons (Figure 3). The inferred exon structure of this gene is different to that of other metazoan aquaporins and the gene appears to be a hybrid with exons 1-3 more related to AQP2 and -5, but exons 4-8 showing higher nucleotide sequence identity to AQP1 and -6 (Additional file 3). Using BLAST we noted that it was related to tetrapod *AQP5*. To validate the existence of this gene, we isolated genomic DNA based upon the predicted ensembl sequence. Using PCR and subsequent sequencing, we found that exons 1-3 are 100% identical to the predicted sequence, and the gene was therefore named *draqp5/1 *in accordance with its hybrid status. Maximum likelihood analysis of the codons of *draqp5/1 *confirmed that exons 1-3 encode a putative protein that is structurally related to tetrapod AQP5 and -2, while exons 4-8 encode a putative protein that is more related to AQP1 (Additional file 4). Bayesian analysis of the codons placed exon 1-3 basal to *AQP0*, but the protein product as a polytomy between AQP5 and AQP2 (data not shown). It therefore appears that despite retaining a large aquaporin repertoire of which ~40% are duplicate isoforms specific to the teleost crown-clade, the zebrafish lacks functional orthologs of tetrapod *AQP2*, *-5 *or *-6 *genes.

### Phylogenetic analysis of zebrafish aquaporins

**Figure 4:**
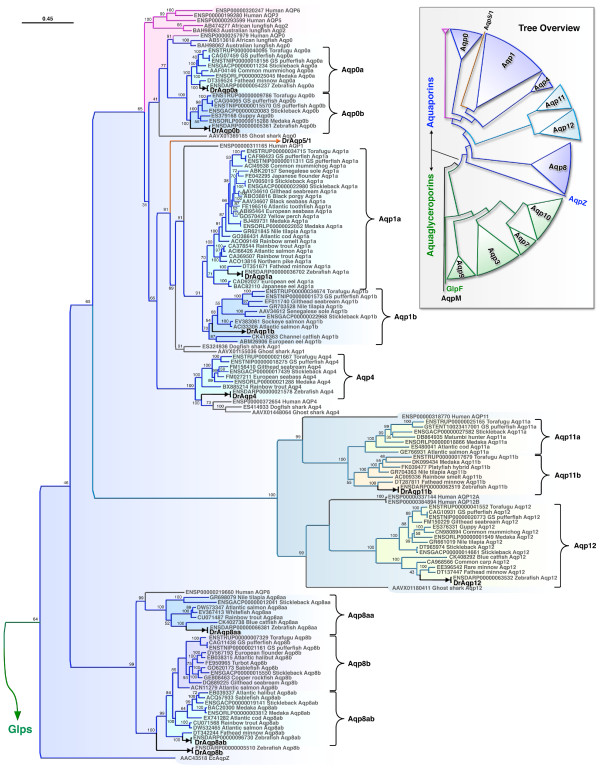
**Bayesian majority rule consensus tree for the codon alignment of piscine and human aquaporins**. The upper right panel shows the summarized topology of the complete tree rooted with archaean *aqpm*. The left panel shows the topology of the classical aquaporins (*aqp0*, *-1*, and *-4*), unorthodox aquaporins (*aqp11 *and *-12*) and Aqp8. Accession numbers are annotated with the taxa. Bayesian posterior probabilities are shown at each node. Scale bar indicates the rate of expected nucleotide substitutions per site.

**Figure 5:**
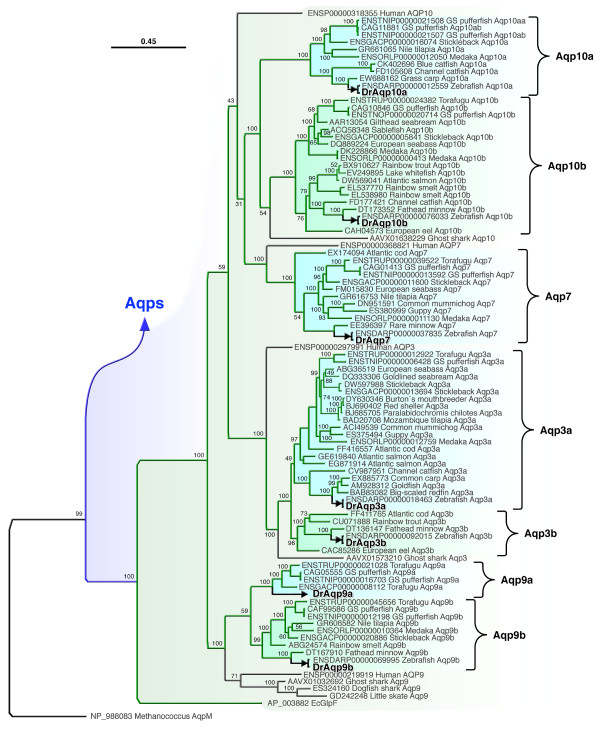
**Bayesian majority rule consensus tree for the codon alignment of piscine and human Glps**. The tree illustrates the expanded topology of the classical Glps (Aqp9, -3, -7 and -10) from the summarized complete tree shown in Figure 4. Accession numbers and other annotations are as described for Figure 4.

To validate the orthology of the zebrafish aquaporins, we investigated the molecular phylogeny of 233 piscine nucleotide and amino acid sequences in relation to 14 human orthologs (Figures 4 and 5). These analyses corroborated the structural homologies outlined above and consistently demonstrated a dual- or in the case of Aqp8 a tri-paralogous clustering of piscine isoforms as sister branches to human orthologs. Two mutually exclusive clades are observed within the superfamily, a vertebrate aquaporin clade containing nine subfamilies (Aqp8, -12, 11, -4, -1, -0, 2, -5 and -6) stemming from EcAqpZ (Figure 4), and a Glp clade containing four vertebrate subfamilies (Aqp9, -3, -7 and -10) that stem from EcGlpF (Figure 5). The elasmobranch aquaporins are represented by single-copy variants, of which all but Aqp7, -11, -8, -2, -5 and -6 were found. Each clustered basal to Teleostei or together with the human transcripts indicating that the encoded proteins have differentially evolved functions specific to the actinopterygian or sarcopterygian lineages, respectively. The internal distribution of teleost isoforms within each subfamily was consistent with a whole genome duplication (WGD) event at the root of the crown-clade [20,33-36]. The topology within each teleost subcluster was fully congruent with phylogenetic rank and encompasses members of the Elopomorpha (e.g. eels), Ostariophysi (e.g. zebrafish and carps), Protacanthopterygii (e.g. salmonids) and Acanthomorpha (e.g Gadiformes and Perciformes). We therefore annotated each of the teleost paralogs with the postscript "a" or "b" to reflect the genomic duplicate.

By including genomic variants together with transcripts available from GenBank [37], and by investigating the syntenic relationships of each ortholog (Finn and Cerdà, unpublished data), we were able to validate whether a duplicated gene arose through WGD or resulted from intrachromosomal duplication. Despite the observation that *draqp1a *and *-1b*, *draqp0a *and *-0b*, and *draqp7 *and *-3b *are linked (Figure 3), we only find strong evidence of one tandem duplicate within the crown-clade: *aqp8aa *(ENSDARG00000045141) and *aqp8ab *(ENSDARG00000071592) in zebrafish, and *aqp8aa *(ENSGACG00000009127) and *aqp8ab *(ENSGACG00000014505) in stickleback. One lineage-specific tandem duplicate of *aqp10a *(*aqp10aa*: ENSTNIG00000018340 and *aqp10ab: *ENSTNIG00000018339) was also noted in green-spotted pufferfish (*Tetraodon nigroviridis*). Both *aqp8aa*, *-8ab *and *aqp10aa*, *-10ab *genes are immediately juxtaposed in the genomes of zebrafish (linkage group [LG] 12) and green-spotted pufferfish (LG8), while the WGD products *aqp8b *and *aqp10b *are each located on separate chromosomes, LG3 and LG3, respectively. Unfortunately, it was not possible to characterize the complete *aqp8 *loci in other teleosts due to the current location of stickleback *aqp8ab *on scaffold 211, and the absence of *aqp8aa*, *-8ab *in green-sptotted pufferfish, and *aqp8aa *and *-8b *in medaka (*Oryzias latipes*). For medaka, only the *aqp8ab *gene was found on LG19. However, by comparing our data with the earlier study of Kasahara and colleagues [38], we were able to trace the ancestral chromosome that gave rise to *aqp8 *genes in humans and teleosts. Despite multiple rearrangement, fusion and fission events, the major portions of all descendent LGs that harbour vertebrate *aqp8 *genes can be traced to protochromsome e. For humans this entails LG16, while for Teleostei, the descendent chromosomes are LG2 (predicted locus of *aqp8aa, -8ab*) and LG3 (*aqp8b*) for green-spotted pufferfish, LG19 (*aqp8ab*) and LG1 or LG8 (predicted locus of *aqp8b*) for medaka. The chromosomal loci of *aqp8 *genes in stickleback and zebrafish further support this ancient origin. Moreover, the early divergence of teleost *aqp8aa*, *-8ab *and *-8b *is clearly evident in the trichotomous clustering of these transcripts among diverse taxa (Figure 4), and further confirmed through functional and expression analyses in zebrafish (see below).

Similar ancestral reconstructions can be made for the other linked aquaporins, wherein *aqp1 *genes descend from protochromosome m, *aqp0 *originated on prototochromosome l, and *aqp7 and -3 *derive from protochromosomes l and i, respectively. In this latter instance, *aqp7 *and *-3 *have remained linked in all vertebrata, except the opossum (*Monodelphis domestica*), and thus likely became colocalized prior to the separation of Sarcopterygii from Actinopterygii. The evidence supporting this latter proposal lies in the observation that zebrafish has maintained the linkage between *aqp3b *and *-7 *on LG21, while *aqp3a *is located on LG5. Zebrafish LG5 is the orthologon of stickleback LGXIII and medaka LG9, both of which harbour the *aqp3a *orthologs. Hence the single *aqp7 *gene found in teleost genomes is the surviving *aqp7b *product of WGD. The close linkage of teleost *aqp1a *and *-1b *genes, suggests that they could have arisen through tandem duplication. However, the fact that all teleost genomes, except medaka, retain both isoforms, and that each shows dichotomous clustering in the phylogenetic trees (Figure 4), their origin must be close to, or coincident with the WGD event at the root of the crown-clade. Hence, although we cannot exclude a tandem duplication scenario suggested earlier [22], a parsimonious explanation for the appearance or *aqp1a *and *-1b *would seem to be WGD with subsequent colocalization, rather than local duplication and loss of WGD paralogs. The divergence of these isoforms is clearly evident in the phylogenetic trees, where *aqp1b *transcripts display significantly longer branch lengths compared to the *aqp1a *paralogs. This latter feature is consistent with our earlier findings wherein Aqp1b is a rapidly evolving channel protein with novel functions specifically associated with oocyte hydration [17-22] and osmoregulatory processes [16].

In a separate analysis of metazoan aquaporins (Finn and Cerdà, unpublished data) we confirmed that *aqp7, -12 *and *-4 *have remained single-copy genes in non-human vertebrates while the present data show that *Aqp11 *duplicated at the root of the teleost crown-clade. With the exception of torafugu (*Takifugu rubripes*), which retains both *aqp11a *and *-11b*, Teleostei, including zebrafish, appear to have differentially retained alternate isoforms. This is clearly shown in the phylogenetic tree (Figure 4), with closely related Protacanthopterygii and Acanthomorpha harbouring opposite isoforms. Based upon the more ancestral ostariophysan position of zebrafish and the fathead minnow (*Pimephales promelas*), we arbitrarily assigned the zebrafish cluster as the "b" variant.

Although zebrafish retains two isoforms of *aqp3*, these genes proved the most difficult class of Glp to resolve (Figure 5). Here we show the codon topology, which also includes variants obtained from EST databases. By specifically increasing the taxon sampling of these Glps, we were able to resolve a branch topology that matched the chromosomal loci of the teleost genes. As a result, the genomic duplicates are annotated as "a" or "b" accordingly. However, the protein trees did not always corroborate the codon topology and therefore these Glps will require further validation with the advent of new sequence data. The placement of *draqp5/1 *as an outgroup between *aqp0 *and *-1 *was consistent with its hybrid status, while the *AQP2*, *-5 *and *-6 *genes are specific to the sarcopterygian lineage.

### Molecular features of zebrafish aquaporins and functional implications

**Figure 6:**
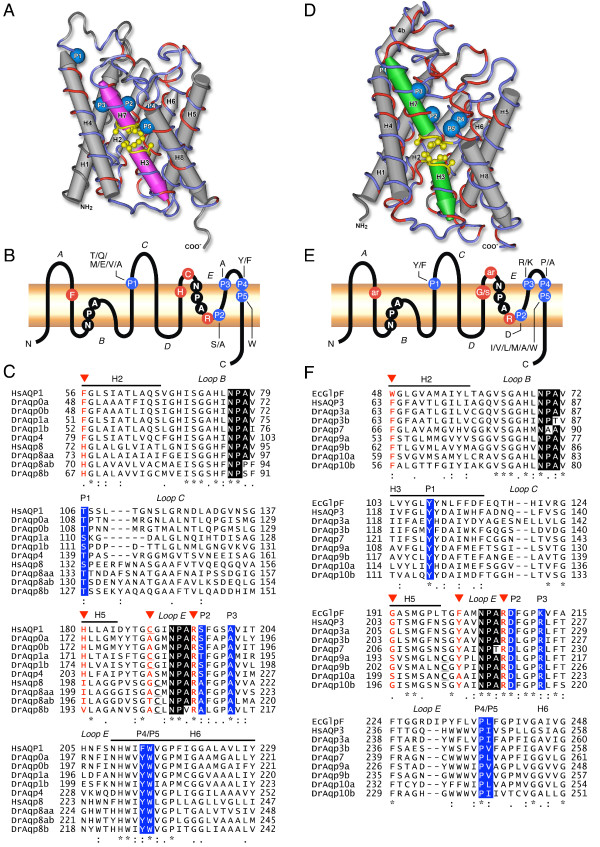
**Structural features of zebrafish aquaporins**. (A-C) Water-selective aquaporins and aquaglyceroporins (Glps; D-F). (A and D) Three-dimensional reconstruction of DrAqp4 wrapped to the crystallographically resolved structure of *Escherichia coli *AqpZ (1RC2 chain B), and DrAqp3a wrapped to the crystallographically resolved structure of *E. coli *GlpF (1LDI chain A). Molecules are mirror tube-worm renders rotated to show identical (red) and non-identical (blue) residues and the annotated features including the blue space-filled conserved sites (P1-P5) and the opposing yellow ball and stick Asn-Pro-Ala (NPA) motifs between hemi-helices H3 and H7. Despite low primary identity/similarity (21.1/36.6% for DrAqp4; 34.1/53.0% for DrAqp3a) the secondary and tertiary structures appear conserved. (B and E) Schematic diagram of aquaporin monomers showing the 6 transmembrane helices (H), the two NPA motifs, the amino acids forming the aromatic/arginine (ar/R) constriction, and the five residues (P1-P5) conserved in water-selective (B) and Glps (E). In each position the conserved residues are indicated. (C) Amino acid sequence alignment of human AQP1 and AQP8 (HsAQP1 and HsAQP8), mouse AQP8 (MmAQP8), DrAqp0a, -0b, -1a, -1b, -4, and zebrafish AQP8-related sequences (DrAqp8aa, -8ab and -8b). The arrowheads point to the positions of the ar/R constriction. The P1-P5 conserved amino acids are shaded in blue. The asterisks indicate identical residues, whereas conserved amino acid substitutions and substitutions with similar amino acids are indicated by a double or single dot, respectively. The potential mercury-sensitive Cys site before the second NPA motif is underlined. (F) Amino acid sequence alignment of EcGlpF, human AQP3 (HsAQP3) and zebrafish Glps. Symbols and notes as in C.

The characteristic residues in a water channel that distinguish a true water facilitator from a Glp have previously been analyzed by comparing sequences of aquaporins with known functions [39]. That comparison resulted in the identification of five invariant or nearly invariant residues (P1 to P5) in aquaporins and Glps on the basis of 153 sequences ranging from bacteria to humans (Figure 6A,B,D,E). However, structural and functional studies of AQP1 and GlpF suggest the presence of two constriction sites in the water pore, in addition to the P1-P5 residues, that underlie their high selectivity and efficiency with regard to water or glycerol transport [9,40-43] (Figure 1).

The first constriction is formed by the opposing NPA motifs located at each positive end of α-helices 3 and 7 (on loops B and E; Figure 6A,B,D,E), such that the Asn creates an electrostatic barrier in this region [44]. Together with desolvation, these regions are essential for water transport specificity while excluding proton transport [44]. Accordingly, Asn is the least variable in aquaporin sequences whereas Pro and Ala are more exchangeable [2,45]. The primary structure of zebrafish aquaporins and Glps confirm these observations since Asn was conserved in both NPA boxes of all paralogs, whereas Pro is substituted by Ala in the first box of DrAqp7 as observed in human and rat AQP7 (Figure 6F). The third residue of the NPA boxes shows more variation as previously found in over 450 aquaporin-encoding genes [2]. In zebrafish, Ala is substituted by Pro (first box of DrAqp8ab), Ser (first box in DrAqp8b), Val (second box in DrAqp8b), or Thr (first and second box in DrAqp3b, and DrAqp7 and -11b, respectively).

The second significant energy-barrier in aquaporins is located close to the extracellular exit of the channel forming the narrowest region of the pore and is referred to as the aromatic/arginine (ar/R) constriction [46,47]. This region in water-selective aquaporins is formed by four amino acids (Phe^56^, His^180^, Cys^189 ^and Arg^195 ^in human AQP1; Figure 6B,C,E,F) that create the hydrophobic and size filter [48]. The site sensitive to mercurial inhibition (Cys^189 ^in AQP1) [11] is less conserved. In contrast, in GlpF, and essentially in all other Glps, the ar/R region is wider and more hydrophobic due to the lack of His and substitution of the Cys by a second aromatic residue, which allows the passage of polyols and urea, and possibly of other small solutes such as NH_3_, CO_2 _or O_2 _[48].

The present data show that zebrafish aquaporins previously inferred to be water-selective or Glps after amino acid sequence analysis retain the respective three amino acid consensus in the ar/R constriction, and show the P1-P5 residues conserved in each sub-family (Figure 6 C,F). However, the Cys residue just prior to the second NPA box, which is potentially mercury-sensitive, is maintained in DrAqp1a and -1b. Similarly, DrAqp8aa, -8ab, -8b, -9a, -9b and -10a also show a Cys upstream of the second NPA motif in loop B but at slightly different positions. Despite these minor differences, the structural features of zebrafish aquaporins strongly suggest that they encode functional channels. A slightly different situation is found in DrAqp8aa, -8ab and -8b, which despite having the conserved P1-P5 residues of the aquaporin subfamily, contain His instead of Phe^56^, and Val or Ile instead of His^180^, in the ar/R constriction (Figure 6C). As for their mammalian counterparts, DrAqp11b and -12 appear to be more divergent since they did not show any of the typical residues in the ar/R constriction and only three aquaporin-conserved amino acids (P2, P4 and P5) (data not shown).

### Water and solute permeability of zebrafish aquaporins

Typically, aquaporin water-channel activity is tested by the oocyte-swelling assay [3], in which *Xenopus laevis *oocytes expressing aquaporins are exposed to hypo-osmotic shock and the subsequent water influx is measured by determining volume changes over time. Solute permeability can also be determined volumetrically in isotonic solutions, or by employing radiolabeled compounds [49,50]. Therefore, to determine experimentally the substrate specificity of the zebrafish aquaporins, the cloned cDNAs were expressed in *X. laevis *oocytes.

**Figure 7:**
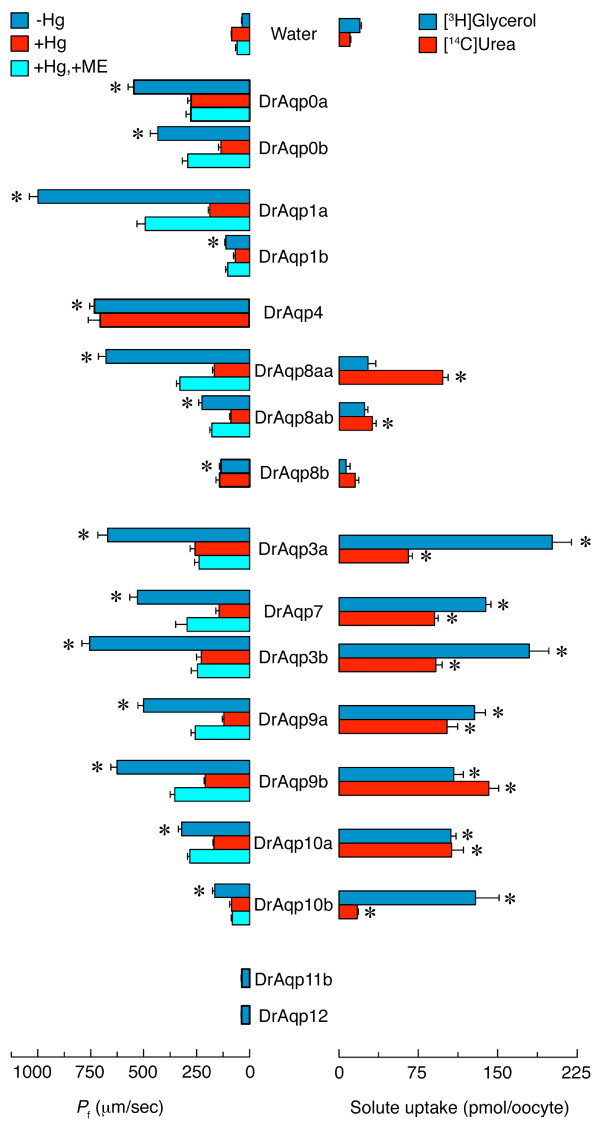
**Functional characterization of zebrafish aquaporins**. Osmotic water permeability (*P*_f_; left), and glycerol and urea uptake (right), of *Xenopus laevis *oocytes expressing zebrafish aquaporins. The *P*_f _was assayed in the presence or absence of HgCl_2 _and β-mercaptoethanol (ME). Values (mean ± SEM; *n *= 8-10 oocytes) with an asterisk are significantly (*p *< 0.01) different from water-injected oocytes in a representative experiment.

Expression of all of the aquaporin isoforms, except *draqp11b *and *-12*, induced a three- to twenty-fold increase in oocyte water permeability (Figure 7). Interestingly, however, water permeability of oocytes expressing *draqp3a *and *-3b *was reduced by acidic pH, as found for mammalian AQP3 [51] and European eel (*Anguilla anguilla*) Aqp3 [52], with maximum permeability occurring at pH 8.5. Swelling (data not shown) and isotope-labeled solute uptake assays demonstrated that oocytes injected with *draqp3a*, *-3b*, *-7*, *-9a*, *-9b*, *-10a *and *-10b *were also permeable to glycerol and urea, although in the case of *draqp10b *urea permeability was low (Figure 7). Oocytes expressing *draqp8aa *or -*8ab *were permeable to water and urea, but not glycerol. In contrast, oocytes expressing *draqp0a*, *-0b*, *-1a*, *-1b *and *-4*, as well as *draqp8b*, were not permeable to either of these solutes.

Water and glycerol transport through most aquaporins was significantly (*p *< 0.01) blocked by 0.3 mM HgCl_2 _even in the absence of Cys^189 ^in their deduced amino acid sequences (Figure 6C,F). Such inhibition was not always reversed with the reducing agent β-mercaptoethanol. However, DrAqp4 (as in mammalian AQP4) and DrAqp8b were mercury-insensitive, even though DrAqp8b has a potential mercury-sensitive Cys upstream of the second NPA box (Figure 6C). It thus appears that water and solute flux through zebrafish aquaporins can be blocked by mercurial compounds regardless of the presence or absence of Cys^189^. These observations have also been noted for rat AQP3 [53,54] and plant aquaporins [55,56]. The present findings suggest that sensitivity of zebrafish aquaporins to mercurials is a complex phenomenon, as has been suggested for other aquaporins, and may involve other residues in addition to Cys^189 ^[56].

Expression of *draqp11b *or *-12 *had no effect on oocyte water (Figure 7), or glycerol (data not shown) permeability. The absence of water and solute transport in *X. laevis *oocytes expressing mammalian AQP11 and -12 has been previously reported [57,58]. For AQP12, this seems to be caused by the absence of protein expression in the oocyte plasma membrane [57], but in the case of AQP11 the protein is readily targeted to the plasma membrane [58]. The underlying mechanisms involved in the functional failure of AQP11 and -12 in oocytes are not well known, but may be related to the fact that these aquaporins are localized intracellularly *in vivo *[57,59]. However, when AQP11 was reconstituted into liposomes this protein proved to be a functional water channel [60]. Although the identity of DrAqp11b and -12 with AQP11 and -12 is low (35% and 38% identity, respectively), a similar situation may be speculated in zebrafish. Further studies employing reconstitution of DrAqp11b and -12 into proteoliposomes, as well as specific antibodies to elucidate their subcellular localization, would help to clarify this issue.

### Expression pattern of zebrafish aquaporins

**Figure 8:**
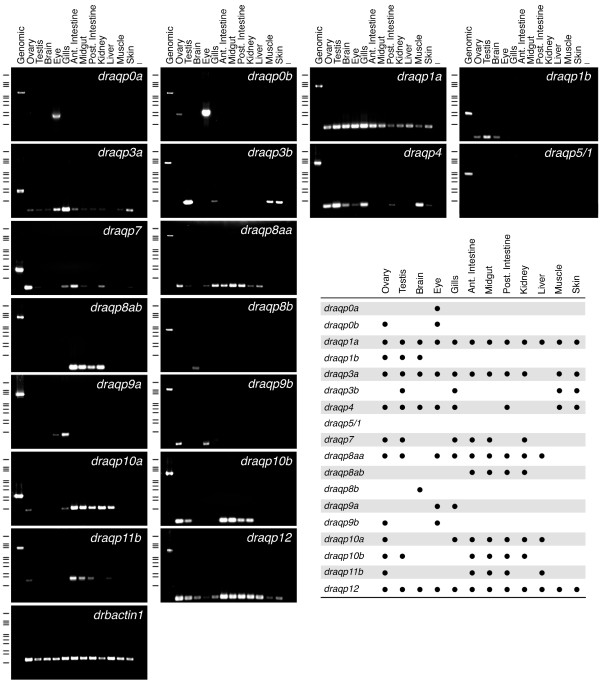
**Aquaporin gene expression in adult tissues of zebrafish**. Representative RT-PCR analysis of aquaporin and *b-actin1 *(*drbactin1*) transcripts. PCR on genomic DNA was used as control. Minus indicates absence of RT during cDNA synthesis. The size (kb) of PCR products and molecular markers are indicated on the left; from top to bottom: 21.23, 5.15, 4.27, 3.53, 2.03, 1.91, 1.58, 1.37, 0.95, 0.83 and 0.56. A summary of the presence or absence of the aquaporin transcripts in the different tissues is shown to the lower right.

The relative expression of the zebrafish aquaporin genes in adult tissues was evaluated by RT-PCR employing isoform-specific oligonucleotide primers (Figure 8). These data revealed that while all genes except *draqp5/1 *were expressed, some aquaporin transcripts were ubiquitiously distributed in the tissues examined or appeared to be tissue-specific. Notably, mRNAs derived from duplicated paralogs showed slightly different expression patterns, although redundancy in some tissues was also observed.

Only two zebrafish aquaporin transcripts (*draqp1a *and *-12*) showed tissue-wide expression patterns. The ubiquitous expression of *draqp1a *transcripts is consistent with the presence of the mammalian AQP1 ortholog in endothelial barriers of almost all tissues and in many epithelia [61]. Its presence in the zebrafish gastrointestinal tract and gills agrees with that reported in other teleosts [12,15,16,62]. In contrast, expression of *draqp1b *was restricted to the ovary, testis and brain, as previously reported [22]. The ubiquitous expression of the unorthodox aquaporin *draqp12*, with lower levels in the eye and muscle, differs markedly from the exclusive expression of mammalian AQP12 in the pancreatic acinar cells [57] and the retina [63]. The vertebrate-specific unorthodox aquaporin, *draqp11b*, also showed a different expression pattern compared to that described for mammalian AQP11. In mice, kidney expression of AQP11 seems to be essential during development since either its absence or mutations of the amino acid sequence induce renal failure [59,64]. In our PCR experiments, however, we could not observe detectable levels of *draqp11b *transcripts in the kidney, although they were abundantly expressed in the gut and were also detected in ovary and liver.

In contrast to *draqp1a *and *-12*, transcripts of *draqp0a *and *-8b *were exclusively detected in tissues of the eye and brain, respectively, and *draqp0b *was also observed in the ovary. The specific expression of *draqp0 *paralogs in the eye is consistent with that previously described in the common mummichog (*Fundulus heteroclitus*) [65,66], as well as in mammals where AQP0 is predominantly present in fibre cells of the lens [67]. Expression of *draqp8b *was noted only in the brain, where *draqp8aa *and *-8ab *were not expressed. In contrast, these latter transcripts were abundantly expressed in the gut and kidney, *draqp8ab *mRNA being also present in the ovary, testis, eye, gills and liver. The diffuse expression of *draqp8aa *and *-8ab *resembles that found for mammalian AQP8, which is present in the testis, ovary, kidney, liver, pancreas, small intestine and colon [1,68]. The expression of *draqp4 *is similar to that of mammalian AQP4 [1], which is distributed in the brain, small intestine and muscle, but also in kidney. In the present study, however, we did not detect *draqp4 *transcripts in the zebrafish kidney.

The distribution of classical Glp mRNAs in zebrafish was comparable to that described for mammals and some teleosts [69,70]. *draqp3a *showed the most extensive distribution in all tissues examined except in the liver, showing high abundance in gills as previously found for other teleosts [14,15]. In contrast, *draqp3b *transcripts were seen only in the testis, gills, muscle and skin. The *draqp9a *and *-9b *isoforms, as well as *draqp10a *and *-10b*, also showed differential expression in some tissues. The expression of two functional isoforms of *draqp10 *in zebrafish, however, differs from that reported for mouse in which AQP10 was suggested to be a pseudogene [71]. As in mammals, *draqp7 *is expressed in the gonads and the kidney, while the expression observed in the intestine may reflect an adipocyte function [72,73].

The RT-PCR studies suggest that redundant expression of duplicated aquaporin isoforms occurs in many tissues of zebrafish. The physiological significance of these findings is intriguing. In other teleosts, such as the gilthead seabream (*Sparus aurata*), redundant expression of *aqp1a *(*saaqp1a*) and *-1b *(*saaqp1b*) in the ovary and gastrointestinal tract is also observed, but immunological studies have revealed that their protein products are differentially localized [16,17]. Thus, in the ovary SaAqp1b is oocyte-specific, whereas SaAqp1a seems to be only present in the external epithelium surrounding the ovarian follicle. In the gut, SaAqp1b is exclusively detected at the apical membrane of rectal enterocytes, whereas SaAqp1a is localized at the brush border of enterocytes in the duodenum and hindgut. Therefore, the significance of the redundant aquaporin expression in zebrafish must be further investigated by determining the subcellular sites of transcription and protein targeting.

## Conclusions

In this study, we identified a large number of MIP encoding genes in zebrafish when compared to tetrapods. By integrating the molecular phylogeny and the genomic loci with structural and functional analyses we demonstrate dual- or tri-paralogy between teleost aquaporins and human orthologs. Consequently teleost aquaporins can be classified into the same water-selective and Glp subfamilies previously described in vertebrates [2]. Expression in *X. laevis *oocytes also demonstrated that zebrafish aquaporin genes, except *aqp11b *and *-12*, encode functional channels, which in most cases have retained the substrate specificity of the tetrapod counterparts. Some mammalian aquaporins are also permeable to NH_3_, CO_2 _or O_2 _[74], but whether any of the zebrafish orthologs are permeable to these compounds remains to be investigated.

The high number of aquaporin genes in zebrafish and other teleosts result from WGD at the root of the crown-clade. Most duplicated isoforms are retained, while *aqp4*, *-7*, and *-12 *have remained single-copy genes in non-human vertebrates. The findings further reveal that tandem duplication has occurred within the clade and in a lineage-specific manner, but such intrachromosomal replication events are rare for this superfamily. While most of the duplicated genes seem to be conserved during the diversification of teleosts, some such as *aqp11, -3 and -8 *appear to have been differentially retained among species. The reasons for the evolutionary selection of specific aquaporin isoforms in teleosts, as well as the potential neofunctionalization of others, as shown for *aqp1b *in the hydrating oocytes of marine teleosts [17,22], have yet to be determined.

An interesting finding was that zebrafish harbours a hybrid gene with partial structural identity to the tetrapod *AQP2*, *AQP5 *and *AQP6 *orthologs. This hybrid sequence is not expressed, and it appears to represent a pseudogene. The physiological significance of this observation remains to be elucidated. The present study, however, provides a validated nomenclature for the piscine aquaporin superfamily and lays the foundation for further functional studies in zebrafish.

## Methods

### Fish

Adult zebrafish were purchased from local pet stores and maintained as described [75]. To obtain samples of the different tissues, fish were sedated by immersion for approximately 15 min in 80-100 ppm phenoxyethanol, and sacrificed by decapitation. Tissue samples were immediately processed for RNA extraction or flash-frozen in liquid nitrogen and stored at -80°C. Procedures relating to the care and use of animals were approved by the Ethics Committee from IRTA in accordance with the Guiding Principles for the Care and Use of Laboratory Animals.

### Genome and transcript analysis

Zebrafish aquaporin coding sequences were identified by basic local alignment search tool (BLAST) [76] homology searches of the NCBI [37] and ensembl [32] databases with mammalian and previously cloned teleost aquaporin sequences. The predicted amino acid sequences were extracted, analyzed using BLASTP, and annotated according to the nomenclature established for zebrafish [30]. Intron-exon splicing sites were verified visually using available cDNA sequences and/or corresponding ESTs, and genomic sequences. Potential transmembrane helices in the encoded amino acid sequences were predicted using the SOSUI engine v1.11 [77], TMHMM Server v2.0 [78] and Phobius [79], or via molecular wraps to resolved aquaporin structures (Additional file 5).

### cDNA Cloning

In this study, cDNAs bearing the complete coding region of *draqp0a*, *-0b*, *-3a*, *-3b*, *-4*, *-7*, *-8aa*, *-8ab*, *-8b*, *-9a*, *-9b*, *-10a*, *-10b*, *-11b*, and *-12 *were cloned by conventional RT-PCR. Full-length *draqp1a *and *-1b *cDNAs were previously isolated [17,22]. Total RNA was extracted from the kidney, gut, gills, liver, brain and ovary of adult fish using the RNeasy Maxikit (Qiagen) followed by DNase treatment following the manufacturer's instructions. A pool of 1-10 μg total RNA from the different tissues was reverse transcribed using 0.5 μg oligo (dT)_17_, 1 mM dNTPs, 40 IU RNAse inhibitor (Roche), and 10 IU MMLuV-RT enzyme (Roche), for 1.5 h at 42°C. For amplification, PCR was carried out with 1 μl of the synthesized cDNA and specific oligonucleotide primers designed according to the available cDNAs and genomic sequences. The PCR reactions were conducted using a total volume of 50 μl containing 1x reaction buffer supplemented with 1.5 mM MgCl_2_, 0.2 mM dNTPs, 0.2 mM of each forward and reverse primer, and 1U of Easy-ATM High-Fidelity PCR Cloning Enzyme (Stratagene). The PCR conditions were an initial denaturation at 95°C for 5 min, followed by 36 cycles at 95°C for 1 min, 60-65°C for 1 min (depending on the Tm of each primer pair), and 72°C for 1 min. A final elongation cycle at 72°C for 7 min was carried out in all cases. The products were cloned into the pGEM-T Easy Vector (Promega) and both strands sequenced by BigDye Terminator v3.1 cycle sequencing on ABI PRISM 377 DNA analyzer (Applied Biosystems). The deduced amino acid sequences of cloned cDNAs were 100% identical to those available in the databases, except for DrAqp7 and -11b, which were 98% identical (3 and 2 amino acid changes, respectively), and the cDNAs encoding DrAqp8aa, -8ab, -9b, -10a and -12, which were 99% identical (1 amino acid change in each sequence). In further structural and phylogenetic analyses we used the amino acid sequences derived from the cDNAs cloned in this study since they were found to encode functional proteins in oocytes.

### Functional expression in *Xenopus laevis *oocytes

Zebrafish aquaporin full-length cDNAs were cloned into the *Eco*RV/*Spe*I sites of the oocyte expression vector pT7Ts [80]. Capped RNAs (cRNAs) were synthesized *in vitro *with T7 RNA Polymerase (Roche) from *Xba*I-linearized pT7Ts vector containing the different aquaporin cDNAs. The isolation and microinjection of stage V-VI oocytes was performed as described previously [80]. Oocytes were injected with 50 nl of distilled water (negative control) or 50 nl of water solution containing 1-10 ng cRNA.

### Swelling assays

The osmotic water permeability (*P*_f_) was measured from the time course of oocyte swelling in a standard assay. Oocytes were transferred from 200 mOsm modified Barth's culture medium (MBS; 0.33 mM Ca(NO_3_)_2_, 0.4 mM CaCl_2_, 88 mM NaCl, 1 mM KCl, 2.4 mM NaHCO_3_, 10 mM Hepes, 0.82 mM MgSO_4_, pH 7.5) to 20 mOsm MBS at room temperature. Oocyte swelling was followed by video microscopy using serial images at 2 s intervals during the first 20 s period. For Draqp3a and -3b, the swelling assays were performed at pH 8.5. The *P*_f _values were calculated taking into account the time-course changes in relative oocyte volume [d(V/V_o_)/dt], the molar volume of water (V_w _= 18 cm^3^/ml) and the oocyte surface area (S) using the formula V_o_[d(V/V_o_)/dt]/[SV_w_(Osm_in _- Osm_out_)]. To examine the inhibitory effect of mercury on *P*_f_, oocytes were pre-incubated for 15 min in MBS containing 0.5 or 0.3 mM HgCl_2 _before and during the swelling assays. To determine the reversibility of the inhibition, the oocytes were rinsed 3 times with fresh MBS and incubated for another 15 min with 5 mM β-mercaptoethanol before being subjected to swelling assays.

### Radioactive solute uptake assays

To determine the uptake of [^3^H]glycerol (60 Ci/mmol) and [^14^C]urea (52 mCi/mmol) groups of 10 oocytes, injected with water or cRNA, were incubated in 200 μl of MBS containing 20 μCi of the radiolabeled solute (cold solute was added to give 1 mM final concentration) at room temperature. After 10 min (including zero time for subtraction of the signal from externally bound solute), oocytes were washed rapidly in ice-cold MBS three times, and individual oocytes were dissolved in 5% SDS for scintillation counting.

### Statistical analysis of *P*_f _and solute uptake

Data are expressed as mean ± SEM. The data shown are from a representative experiment out of 3-4 different trials producing similar results. The measured values of *P*_f_, and glycerol and urea uptake were statistically analyzed in an unpaired Student's *t *test; *p *values < 0.01 were considered significantly different.

### Gene expression analysis

Total RNA was extracted from different tissues, ovary, testis, brain, eye, gills, anterior intestine, midgut, posterior intestine, kidney, liver, muscle and skin, of 5-10 adult fish using the RNeasy Mini Kit (Qiagen). An aliquot of RNA (500 ng) was treated with Turbo-DNase (Ambion) and reverse transcribed as described above. PCR was carried out with 0.3-0.6 μl of cDNA employing *Tfi *DNA Polymerase (Invitrogen) and aquaporin isoform-specific oligonucleotide primers (Additional file 6). For each aquaporin nucleotide sequence, the oligos were designed to flank one or separated introns that were complementary to non-conserved regions among aquaporin paralogs. The reactions were carried out in a 25 μl volume containing 1x reaction buffer, 1.5 mM MgCl_2_, 0.2 mM dNTPs, 0.2 mM of each primer and 1U of *Tfi *polymerase. The PCR conditions were as described above except for the number of the cycles, which was increased to 40. As a reference gene to control the variation in mRNA concentration, zebrafish *b-actin1 *(*drbactin1*) was used, employing PCR conditions of 22 cycles at 95°C for 30 s, 55°C for 1 min, and 72°C for 1 min. Amplification of genomic DNA (500 ng), purified from liver, was used as a positive control using the same PCR conditions but with an elongation step of 1 min per kb. An aliquot of the PCR reactions was electrophoresed on 1% agarose gels containing ethidium bromide and the products were visualized and photographed.

### Phylogenetic analysis

Orthologs of the aquaporin superfamily were obtained from public GenBanks via entrez and BLAST and identified via BLASTP, BLASTN or BLAT from ensembl [32] and the ghost shark (*Callorhinchus milii*) [81] genome databases. Construction of aquaporin amino acid multiple sequence alignments was achieved using the t-coffee v7.54 suite of tools [82] and ClustalX [83]. Each amino acid alignment was converted to a codon alignment (nucleotide triplets) as described previously [20] and manually adjusted to correct errors using MacVector (MacVector Inc, Cambridge, UK). Three-dimensional alignments of each zebrafish amino acid sequence against crystallographically resolved aquaporin molecules (Additional file 5) were used to identify conserved secondary structures in order to minimize gaps in the α-helical regions. Three-dimensional protein wraps were rendered using Cn3D [37] as described previously [24]. Identity and similarity matrices were calculated using MacVector.

Preliminary Bayesian analyses (Mr Bayes v3.1.2; [84]) were run for all sequences, and identical predictions pruned from the alignments. Where possible GenBank sequences were run with predicted variants to validate the latter. Prior to phylogenetic analyses, the alignments were trimmed to remove unrelated N- and C-terminal regions. To determine the influence of lesser-conserved regions, large gap regions were removed and these alignments tested via Bayesian analyses. This resulted in shorter branch lengths, and an increased incidence of polytomies, but no significant change to the tree topologies.

Each data set was modeled primarily via Bayesian, but also via maximum likelihood or maximum parsimony methods of phylogenetic inference as described previously [20]. Neighbor joining (NJ) methods were used for efficient identification of orthologs. Tree topologies were accepted after validating convergence using Tracer [85] and when the codon and protein tree topologies were congruent. For Bayesian analyses the following settings were used for codon alignments: nucmodel = 4by4, nst = 2, rates = gamma; and amino acid alignments: aamodel = mixed, with 1,000,000 generations, sampled every 100 generations using 4 chains and a burnin of 3,500. For each run, a majority rule consensus tree together with posterior probabilities from the last 6,500 trees, representing 650,000 generations was arranged using Archeopteryx [86] and subsequently rendered with Geneious Pro (Biomatters Ltd, Auckland, New Zealand). Maximum likelihood codon trees were attained using PAUP v4b10 (Sinauer Associates Inc.) and rendered using FigTree v2.2 [87]. Final trees were annotated with accession numbers using Adobe Photoshop.

### Sequence accession numbers

The zebrafish aquaporin cDNA nucleotide sequences reported in this study, which encoded functional proteins when expressed in *X. laevis *oocytes, have been submitted to the DDBJ/EMBL/GenBank database under the following accession numbers: *draqp0a *(FJ666326), *draqp0b *(FJ655389), *draqp3a *(EU341833), *draqp3b *(EU341832), *draqp4 *(FJ666327), *draqp7 *(FJ655385), *draqp8aa *(FJ655386), *draqp8ab *(EU341834), *draqp8b *(FJ695516), *draqp9a *(FJ655387), *draqp9b *(FJ655387), *draqp10a *(FJ655388), and *draqp10b *(EU341836).

## Abbreviations

AQP: aquaporin; BAC: bacterial artificial chromosome; cRNA: capped RNA; EST: expressed sequence tag; Glp: aquaglyceroporin; LG: linkage group; MIP: major intrinsic protein; NIP: nodulin 26-like intrinsic protein; NJ: Neighbor joining; NPA: aspargine-proline-alanine; *P*_f_: osmotic water permeability; PIP: plasma membrane intrinsic protein; TIP: tonoplast intrinsic proteins; DIP: small and basic intrinsic protein; WGD: whole genome duplication.

## Authors' contributions

ATS, MC and FC carried out the cloning, functional analysis in oocytes and gene expression experiments. RNF, JL and JC analyzed the data and performed structural analyses, and RNF conducted the phylogenetic analyses. JC conceived and coordinated the study, participated in the design of the experiments, and together with RNF wrote the final version of the manuscript. All authors read and approved the final manuscript.

## Supplementary Material

Additional file 1 **Protein sequence identities among zebrafish aquaporins**. The percent identity between zebrafish amino acid sequences. Click here for file

Additional file 2 **Comparison of aquaporin gene structures between zebrafish and other metazoan organisms**. Exon-intron sizes are based on ensembl. In the case of *draqp9a*, the quality of the genomic sequences available did not allow evaluation of the intron sizes. Aquaporin groups are color-coded as described in the key.
Click here for file

Additional file 3 **Identity and similarity scores of zebrafish *aqp5*/1 exons**. Nucleotides and deduced amino acids are compared to tetrapod aquaporins. Data are means ± standard deviations of the sequences submitted to maximum likelihood analysis in Additional file 4. Click here for file

Additional file 4 **Phylogenetic analysis of *draqp5/1***. Maximum likelihood codon trees of zebrafish *draqp5/1 *(ENSDARG00000038202) exons compared to tetrapod orthologs. (a) Exons 4-8; (b) Exons 1-3. Scale bars indicate nucleotide substitution rate. Click here for file

Additional file 5 **PDB structures used in the study**. Crystalographically resolved aquaporin molecules used to optimize the amino acid alignments or identify secondary structures. Click here for file

Additional file 6 **Oligonucleotide primers used for RT-PCR analysis**. Nucleic acid sequences for primers specific for each zebrafish aquaporin mRNA, *bactin1*, and expected product size.
Click here for file
