# Supplementary material for: The zebrafish genome encodes the largest vertebrate repertoire of functional aquaporins with dual paralogy and substrate specificities similar to mammals
Source: BMC Evol Biol. 2010 Feb 11;10:38. doi: 10.1186/1471-2148-10-38 (PMC2829555; doi:10.1186/1471-2148-10-38)
Supplement: Additional file 1 — Protein sequence identities among zebrafish aquaporins. The percent identity between zebrafish amino acid sequences. [file 1471-2148-10-38-S1.PDF]

Protein sequence identities (%) among zebrafish aquaporins

	DrAqp0a	DrAqp0b	DrAqp1a	DrAqp1b	DrAqp4	DrAqp8aa	DrAqp8ab	DrAqp8b	DrAqp11b	DrAqp12	DrAqp10a	DrAqp10b	DrAqp7	DrAqp9a	DrAqp9b	DrAqp3a	DrAqp3b
**DrAqp0a**		**85**	**44**	**45**	**36**	**23**	**24**	**22**	**13**	**15**	**16**	**21**	**20**	**20**	**21**	**23**	**23**
**DrAqp0b**	93		**43**	**44**	**35**	**21**	**25**	**21**	**12**	**16**	**18**	**20**	**20**	**21**	**21**	**24**	**23**
**DrAqp1a**	60	59		**63**	**37**	**22**	**23**	**19**	**14**	**17**	**21**	**22**	**21**	**22**	**23**	**19**	**18**
**DrAqp1b**	62	59	75		**33**	**23**	**24**	**20**	**14**	**14**	**21**	**22**	**22**	**22**	**21**	**19**	**18**
**DrAqp4**	52	50	56	53		**22**	**21**	**20**	**13**	**14**	**14**	**18**	**18**	**20**	**21**	**19**	**18**
**DrAqp8aa**	36	35	40	38	37		**49**	**60**	**14**	**17**	**14**	**18**	**17**	**19**	**19**	**19**	**18**
**DrAqp8ab**	39	38	41	39	39	72		**42**	**12**	**15**	**15**	**19**	**17**	**17**	**17**	**20**	**18**
**DrAqp8b**	35	35	40	38	37	78	66		**16**	**15**	**16**	**17**	**17**	**17**	**18**	**18**	**18**
**DrAqp11b**	26	25	28	28	26	29	29	31		**27**	**12**	**13**	**10**	**13**	**12**	**10**	**11**
**DrAqp12**	29	29	30	25	26	30	30	30	45		**16**	**14**	**16**	**19**	**18**	**14**	**16**
**DrAqp10a**	34	33	40	36	30	34	33	35	26	32		**42**	**42**	**45**	**44**	**39**	**38**
**DrAqp10b**	37	36	37	36	34	32	32	30	23	28	62		**44**	**44**	**43**	**42**	**40**
**DrAqp7**	36	36	38	36	34	33	34	34	27	30	57	59		**41**	**43**	**42**	**42**
**DrAqp9a**	37	36	38	36	34	33	35	31	25	33	63	63	60		**61**	**42**	**43**
**DrAqp9b**	36	35	37	34	33	34	36	35	25	33	61	62	61	78		**43**	**45**
**DrAqp3a**	36	36	34	32	32	33	35	32	23	31	57	61	60	61	61		**73**
**DrAqp3b**	36	35	33	33	32	32	34	32	23	29	56	59	62	64	62	81	
