# Supplementary material for: The zebrafish genome encodes the largest vertebrate repertoire of functional aquaporins with dual paralogy and substrate specificities similar to mammals
Source: BMC Evol Biol. 2010 Feb 11;10:38. doi: 10.1186/1471-2148-10-38 (PMC2829555; doi:10.1186/1471-2148-10-38)
Supplement: Additional file 3 — Identity and similarity scores of zebrafish aqp5/1 exons. Nucleotides and deduced amino acids are compared to tetrapod aquaporins. Data are means ± standard deviations of the sequences submitted to maximum likelihood analysis in Additional file 4. [file 1471-2148-10-38-S3.PDF]

Identity and similarity scores of zebrafish *aqp5/1* exons. Nucleotides and deduced amino acids are compared to tetrapod aquaporins. Data are mean ± standard deviation of the sequences submitted to maximum likelihood analysis in Additional data file 4.

		AQP6	AQP5	AQP2	AQP0	AQP1
exon 1-3:	Codon identity	41 ± 3.0	42 ± 3.4	44 ± 1.8	44 ± 2.1	43 ± 1.6
	Protein identity	30 ± 3.0	36 ± 3.6	38 ± 1.5	33 ± 1.3	32 ± 1.6
	Protein similarity	52 ± 2.1	56 ± 4.5	57 ± 2.0	55 ± 1.9	51 ± 1.5
exon 4-8:	Codon identity	47 ± 1.0	46 ± 2.7	46 ± 1.7	46 ± 1.2	47 ± 1.1
	Protein identity	41 ± 2.5	42 ± 4.1	40 ± 2.0	43 ± 1.4	43 ± 1.6
	Protein similarity	58 ± 3.1	54 ± 4.3	54 ± 1.8	53 ± 1.1	56 ± 1.4
