# Supplementary material for: The zebrafish genome encodes the largest vertebrate repertoire of functional aquaporins with dual paralogy and substrate specificities similar to mammals
Source: BMC Evol Biol. 2010 Feb 11;10:38. doi: 10.1186/1471-2148-10-38 (PMC2829555; doi:10.1186/1471-2148-10-38)
Supplement: Additional file 4 — Phylogenetic analysis of draqp5/1. Maximum likelihood codon trees of zebrafish draqp5/1 (ENSDARG00000038202) exons compared to tetrapod orthologs. (a) Exons 4-8; (b) Exons 1-3. Scale bars indicate nucleotide substitution rate. [file 1471-2148-10-38-S4.PDF]

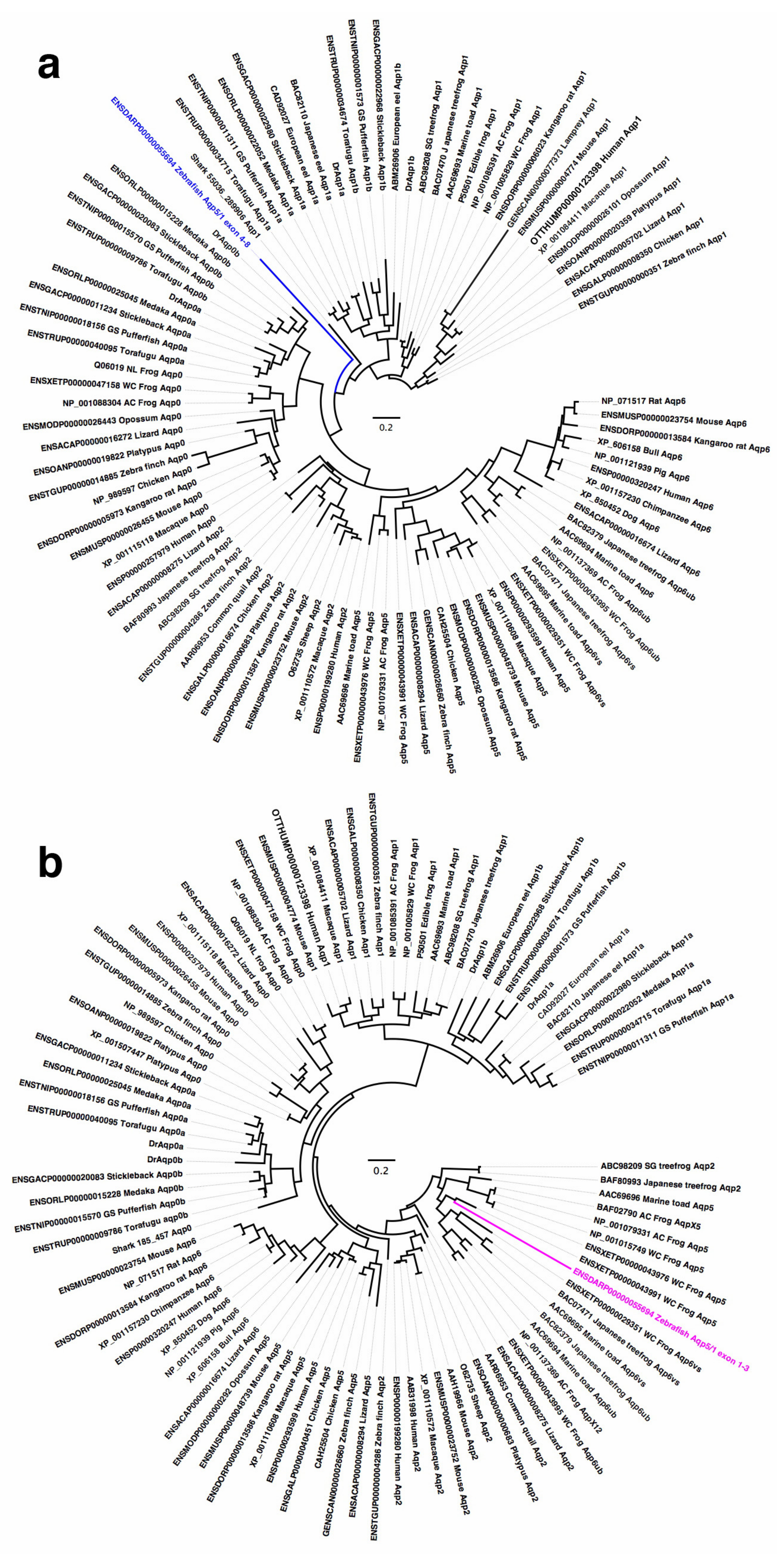
a
ENSDARP00000055694 Zebrafish Aqp5/1 exon 4-8
0.2
b
ENSDARP00000055694 Zebrafish Aqp5/1 exon 1-3
0.2

Maximumim likelihood codon trees of zebrafish *draqp5/1* (ENSDARG00000038202) exons compared to tetrapod orthologs. (a) Exons 4-8; (b) Exons 1-3. Scale bars indicate nucleotide substitution rate.
