# Supplementary material for: The zebrafish genome encodes the largest vertebrate repertoire of functional aquaporins with dual paralogy and substrate specificities similar to mammals
Source: BMC Evol Biol. 2010 Feb 11;10:38. doi: 10.1186/1471-2148-10-38 (PMC2829555; doi:10.1186/1471-2148-10-38)
Supplement: Additional file 5 — PDB structures used in the study. Crystalographically resolved aquaporin molecules used to optimize the amino acid alignments or identify secondary structures. [file 1471-2148-10-38-S5.PDF]

PDB structures used in the study

PDB #	Orthologue	Source			
3D9S	AQP5	Horsefield, R., Norden, K., Fellert, M., Backmark, A., Tornroth-Horsefield, S., Terwisscha van Scheltinga, A.C., Kvassman, J., Kjellbom, P., Johanson, U., Neutze, R.	(2008)	High-resolution x-ray structure of human aquaporin 5	Proc Natl Acad Sci USA 105: 13327-13332
2B6OA	AQP0	Gonen, T., Cheng, Y., Sliz, P., Hiroaki, Y., Fujiyoshi, Y., Harrison, S.C., Walz, T.	(2005)	Lipid-protein interactions in double-layered two-dimensional AQP0 crystals.	Nature 438: 633-638
1YMG	AQP0	Harries, W.E.C., Akhavan, D., Miercke, L.J.W., Khademi, S., Stroud, R.M.	(2004)	The channel architecture of aquaporin 0 at a 2.2-Å resolution	Proc Natl Acad Sci USA 101: 14045-14050
2B6P	AQP0	Gonen, T., Cheng, Y., Sliz, P., Hiroaki, Y., Fujiyoshi, Y., Harrison, S.C., Walz, T.	(2005)	Lipid-protein interactions in double-layered two-dimensional AQP0 crystals.	Nature 438: 633-638
2C32	AQP0	Palanivelu, D.V., Kozono, D.E., Engel, A., Suda, K., Lustig, A., Agre, P., Schirmer, T.	(2006)	Co-axial association of recombinant eye lens aquaporin-0 observed in loosely packed 3D-crystals	J Mol Biol 355: 605-611
1J4N	AQP1	Sui, H., Han, B.G., Lee, J.K., Walian, P., Jap, B.K.	(2001)	Structural basis of water-specific transport through the AQP1 water channel.	Nature 414: 872-878
1H6I	AQP1	De Groot, B.L., Engel, A., Grubmuller, H.	(2001)	A refined structure of human aquaporin 1	FEBS Lett 504: 206
2D57	Aqp4	Hiroaki, Y., Tani, K., Kamegawa, A., Gyobu, N., Nishikawa, K., Suzuki, H., Walz, T., Sasaki, S., Mitsuoka, K., Kimura, K., Mizoguchi, A., Fujiyoshi, Y.	(2005)	Implications of the aquaporin-4 structure on array formation and cell adhesion	J Mol Biol 355: 628-639
2W1P	Aqy1	Fischer, G., Kosinska-Eriksson, U., Aponte-Santamaria, C., Palmgren, M., Geijer, C., Hedfalk, K., Hohmann, S., De Groot, B.L., Neutze, R., Lindkvist-Petersson, K.	(2009)	Crystal Structure of a Yeast Aquaporin at 1.15 Å Reveals a Novel Gating Mechanism	Plos Biol 7: 130
1RC2	AqpZ	Savage, D.F., Egea, P.F., Robles-Colmenares, Y., O`Connell III, J.D., Stroud, R.M.	(2003)	Architecture and selectivity in aquaporins: 2.5 Å X-ray structure of aquaporin Z	Plos Biol 1: 334-340
1LDF	GlpF	Tajkhorshid, E., Nollert, P., Jensen, M.O., Miercke, L.J., O`Connell, J., Stroud, R.M.	(2002)	Control of the selectivity of the aquaporin water channel family by global orientational tuning.	Science 296: 525-530
2EVU	AqpM	Lee, J.K., Kozono, D., Remis, J., Kitagawa, Y., Agre, P., Stroud, R.M.	(2005)	Structural basis for conductance by the archaeal aquaporin AqpM at 1.68 Å.	Proc Natl Acad Sci USA 102: 18932-18937
