# Supplementary material for: The zebrafish genome encodes the largest vertebrate repertoire of functional aquaporins with dual paralogy and substrate specificities similar to mammals
Source: BMC Evol Biol. 2010 Feb 11;10:38. doi: 10.1186/1471-2148-10-38 (PMC2829555; doi:10.1186/1471-2148-10-38)
Supplement: Additional file 6 — Oligonucleotide primers used for RT-PCR analysis. Nucleic acid sequences for primers specific for each zebrafish aquaporin mRNA, bactin1, and expected product size. [file 1471-2148-10-38-S6.PDF]

Oligonucleotide primers used for RT-PCR analysis

Nucleic acid sequences for primers specific for each zebrafish aquaporin mRNA, *bactin1*, and expected size product.

Transcript	GenBank accession number/Ensembl gene ID	Primer sequence 5'-3' (forward/reverse)	PCR size product
draqp0a	FJ666326	TCATCCCTCTGCATTGCTTCTG/ TGCCTCTGGAAGTGTGTGTGTG	816
draqp0b	FJ655389	CGGTTTTGAATTTAGTTGG/ GCGTCGCAGGAGGTTTATT	914
draqp1a	AY626937	GGGATGTGGAATCAATCCTG/ TGAGGTACATACTGATTCGCTGA	455
draqp1b	EU327345	GCCGTTGTCCTCGAGTCTTTT/ CGCTTCGGGGTTCGATTAGT	196
draqp3a	EU341833	AGGCCTTCACAGTGGGATTCAG/ TGAGGGCTGATGGAGATCCTG	339
draqp3b	EU341832	TTTGGACAAGGTTCTCTTGATG/ TCAGAAACGGCAGATAGTTTGA	497
draqp4	FJ666327	CGCCGACCTTGTCCTCATCT/ ATAGCCAGTGCTGCCGAACC	378
draqp5/1	ENSDARG00000038202	CCCACTGCATGTTTCTTTGG/ GGAAACAGTTAATGCACATTTGG	280
draqp7	FJ655385	GCACTGTTGCACAAGTGGTT/ ATTACCCCCGCAGAAATGAT	281
draqp8aa	FJ655386	GCTGCCACTATGGCTGAGATGA/ AAAAGCTTGCAATCCTCTTCA	349
draqp8ab	EU341834	GAGCTGCATTTGCTGTTCTG/ GTGCAGGTCCAAAAGCTCTC	231
draqp8b	FJ695516	GCTGATGGGTTGTTTGTGTG/ CAAGCAGACCTCCAGAGACC	224
draqp9a	FJ655387	CGGGATCCTGTCAGTGACGG/ TCGTATTTGTCCTTCACGCTGC	501
draqp9b	EU341835	TCAATGGCACTAAACTGTGGA/ CCATACATTCCCTCACGGTCT	286
draqp10a	FJ655388	TGCAGACCAATTTCCTGGATCA/ AACGCGGGGAAATAAATCTG	503
draqp10b	EU341836	CGGTTGCCCTGCAGTATTAT/ CCAATGCAAGAACACACACC	182
draqp11b	BC095775	CCCAGCACTGGCATTCTCTA/ AATCAGGGGGAATCAGGAAC	682
draqp12	BC095564	CTGGCCTATGCAGTGACCTTC/ TGTCAGTGTGTTCTGTGTTCGTG	235
drbactin1	NM_131031	ACATGGAGAAGATCTGGC/ GCATACAGGTCCTTACGGA	643
