# Supplementary material for: The zebrafish genome encodes the largest vertebrate repertoire of functional aquaporins with dual paralogy and substrate specificities similar to mammals
Source: BMC Evol Biol. 2010 Feb 11;10:38. doi: 10.1186/1471-2148-10-38 (PMC2829555; doi:10.1186/1471-2148-10-38)
Supplement: Additional file 2 — Comparison of aquaporin gene structures between zebrafish and other metazoan organisms. Exon-intron sizes are based on ensembl. In the case of draqp9a, the quality of the genomic sequences available did not allow evaluation of the intron sizes. Aquaporin groups are color-coded as described in the key. [file 1471-2148-10-38-S2.PDF]

aquaporin 2-5-6 | classical aquaporins | unorthodox aquaporins | classical aquaglyceroporins

Gene	AQP	Chr	Strand	Ex1	In	Ex2	In	Ex3	In	Ex4	In	Ex5	In	Ex6	In	Ex7	In	Ex8	bp
Human (*Homo sapiens*)																			
ENSG00000086159	*AQP6*	12	F	402	748	159	263	81	638	207									2,498
ENSG00000161798	*AQP5*	12	F	363	1,111	165	435	84	816	186									3,160
ENSG00000167580	*AQP2*	12	F	360	2,964	165	310	81	688	210									4,778
ENSG00000135517	*AQP0*	12	R	360	498	165	438	81	1,606	186									3,334
OTTHUMG00000023944	*AQP1*	7	F	384	9,772	165	333	81	805	180									11,720
ENSG00000171885	*AQP4*	18	R	32	3,061	415	886	165	278	81	4,282	279							9,479
ENSG00000103375	*AQP8*	16	F	12	140	248	4,011	127	2,778	215	2,491	135	1,241	49					11,447
ENSG00000178301	*AQP11*	11	F	619	12,944	117	5,625	80											19,385
ENSG00000184945	*AQP12A*	2	R	607	1,762	117	3,607	200											6,293
ENSG00000185176	*AQP12B*	2	F	123	37	448	1,911	117	3,607	199									6,442
ENSG00000143595	*AQP10*	1	F	105	672	127	922	138	121	119	229	218	475	199					3,325
ENSG00000165269	*AQP7*	9	R	141	7,985	124	427	138	208	119	210	218	358	286					10,214
ENSG00000103569	*AQP9*	15	F	111	27,996	127	6,268	138	1,712	119	4,091	218	4,615	175					45,570
ENSG00000165272	*AQP3*	9	R	108	3,530	127	308	138	350	119	333	218	89	169					5,489
Western clawed frog (*Xenopus tropicalis*)																			
ENSXETG00000013389	*AQP6vs*	s167	F	363	2,657	165	857	81	1,677	177									5,977
ENSXETG00000024581	*AQP6ub*	s167	F	363	8,756	165	1,102	81	2,014	180									12,661
ENSXETG00000024580	*AQP5*	s167	F	330	3,852	165	720	81	2,999	114									8,261
ENSXETG00000020388	*AQP2*	s167	F	363	918	162	2,854	81	2,345	216									6,939
ENSXETG00000021804	*AQP0*	s101	F	363	1,313	165	2,436	81	630	186									5,174
ENSXETG00000026986	*AQP1*	s333	F	552	81,919	102													82,573
ENSXETG00000005679	*AQP4*	s83	F	381	1,420	159	2,066	81	947	279									5,333
ENSXETG00000019555	*AQP8*	s255	F	269	426	28	86	96	27	212	111	135	1,144	145					2,679
ENSXETG00000022960	*AQP10*	s1370	F	105	1,222	127	84	138	342	119	2,551	218	93	187					5,186
ENSXETG00000002150	*XAQPt7*	s638	R	108	1,360	127	609	138	1,156	119	313	222							4,152
ENSXETG00000010861	*AQP9*	s308	R	145	8,763	138	1,571	119	894	218	399	166							12,413
ENSXETG00000002151	*AQP3*	s638	R	108	11,203	127	94	138	3,199	119	3,022	129							18,139
ENSXETG00000016307	*AQPU*	s622	F	108	1,072	127	469	138	357	35	692	4	199	65	1	230	76	184	3,573
Zebrafish (*Danio rerio*)																			
ENSDART00000055695	*aqp5/1*	3	F	129	66	223	906	33	778	44	1,931	13	2,756	102	4,948	81	4,180	171	6,981
ENSDARG00000037285	*aqp0a*	23	F	360	106	165	1,504	81	96	186									2,498
ENSDARG00000013963	*aqp0b*	23	F	360	180	165	113	81	1,182	186									2,267
ENSDARG00000023713	*aqp1a*	2	F	357	4,892	165	2,423	81	3,162	180									11,260
novel	*aqp1b*	2	F	366	2,000	165	128	81	736	180									3,656
ENSDARG00000010565	*aqp4*	20	R	32	4,968	421	3,359	165	4,328	81	9,733	264							23,351
ENSDARG00000045141	*aqp8aa*	12	F	263	850	127	890	215	2,922	135	1,259	43							6,704
ENSDARG00000071592	*aqp8ab*	12	F	328	2,048	127	79	215	3,352	135	296	43							6,623
ENSDARG00000015512	*aqp8b*	3	F	245	1,486	127	82	215	72	135	928	43							3,333
ENSDARG00000042617	*aqp11b*	18	F	642	2,329	117	2,943	113											6,144
ENSDARG00000043279	*aqp12*	22	R	290	6,580	343	1,860	117	2,660	128									11,978
ENSDARG00000007086	*aqp10a*	16	F	96	855	127	822	138	1,770	119	243	218	288	205					4,881
ENSDARG00000058678	*aqp10b*	19	F	288	2,000	127	238	89	1,580	119	1,430	218	136	245					6,470
ENSDARG00000026787	*aqp7*	21	F	117	3,380	127	442	138	92	119	3,970	218	743	193					9,539
novel	*aqp9a*																		
ENSDARG00000053480	*aqp9b*	7	F	105	1,300	127	8,190	138	395	119	1,770	218	6,270	259					18,891
ENSDARG00000003808	*aqp3a*	5	F	108	2,290	127	131	138	122	125	484	218	563	175					4,481
ENSDARG00000069518	*aqp3b*	21	F	127	640	138	177	119	2,780	218	71	190							4,460
Fruit fly (*Drosophila melanogaster*)																			
FBtr0088141	*aqp4*	2R	R	16	7,616	179	8,272	354	57	189									16,683
FBtr0079929	*aqp4*	2L	F	197	1,642	167	645	127	4,527	208	804	1,392							9,709
FBtr0088137	*aqp4*	2R	F	30	3,375	71	101	156	61	144	737	423	444	36					5,578
FBtr0072102	*aqp8*	2R	R	74	5,991	550	80	219											6,914
FBtr0072107	*aqp8*	2R	R	17	126	559	336	222											1,260
FBtr0072106	*aqp8*	2R	R	606	52	219													877
FBtr0087766	*aqp12*	2R	R	66	487	181	314	414	60	239									1,761
Nematode (*Caenorhabditis elegans*): original *aqp* nomenclature provided for reference																			
F40F9.9: *aqp-4*	*aqp8*	V	F	54	441	127	57	106	419	280	42	56	54	162	96	37			1,931
K07A1.16: *aqp-9*	*aqp12*	I	F	126	49	153	1,634	73	53	101	45	148	41	121	44	88			2,676
ZK1321.3.1: *aqp-10*	*aqp12*	II	R	93	115	97	88	290	100	133	58	104	46	126					1,931
F32A5.5: *aqp-1*	*glp*	II	R	21	1,274	127	51	127	73	138	45	331	47	181					1,931
Y69E1A.7: *aqp-3*	*glp*	IV	R	154	556	146	471	102	47	265	74	547	47	52					2,461
M02F4.8.1: *aqp-7*	*glp*	X	F	174	149	199	46	119	49	371	51	13							1,171

Exon-intron sizes are based on ensembl v56. In the case of *draqp9a*, the quality of the genomic sequences available did not allow evaluation of the intron sizes. Aquaporin groups are colour-coded as described in the key.
